# The Influence of Negative Pressure and of the Harvesting Site on the Characteristics of Human Adipose Tissue-Derived Stromal Cells from Lipoaspirates

**DOI:** 10.1155/2020/1016231

**Published:** 2020-02-10

**Authors:** Martina Travnickova, Julia Pajorova, Jana Zarubova, Nikola Krocilova, Martin Molitor, Lucie Bacakova

**Affiliations:** ^1^Department of Biomaterials and Tissue Engineering, Institute of Physiology of the Czech Academy of Sciences, Videnska 1083, 142 20 Prague 4, Czech Republic; ^2^Second Faculty of Medicine, Charles University, V Uvalu 84, 150 06 Prague 5, Czech Republic; ^3^Department of Plastic Surgery, Hospital Na Bulovce and First Faculty of Medicine, Charles University, Budinova 67/2, 180 81 Prague 8, Czech Republic

## Abstract

**Background:**

Adipose tissue-derived stromal cells (ADSCs) have great potential for cell-based therapies, including tissue engineering. However, various factors can influence the characteristics of isolated ADSCs.

**Methods:**

We studied the influence of the harvesting site, i.e., inner thigh (*n* = 3), outer thigh (*n* = 3), outer thigh (*n* = 3), outer thigh (

**Results:**

We revealed higher initial cell yields from the outer thigh region than from the abdomen region. Negative pressure did not influence the cell yields from the outer thigh region, whereas the yields from the abdomen region were higher under high negative pressure than under low negative pressure. In the subsequent passage, in general, no significant relationship was identified between the different negative pressure and ADSC characteristics. No significant difference was observed in the characteristics of thigh ADSCs and abdomen ADSCs. Only on day 1, the diameter was significantly bigger in outer thigh ADSCs than in abdomen ADSCs. Moreover, we noted a tendency of thigh ADSCs (i.e., inner thigh+outer thigh) to reach a higher cell number on day 7. *Discussion*. The harvesting site and negative pressure can potentially influence initial cell yields from lipoaspirates. However, for subsequent *in vitro* culturing and for use in tissue engineering, it seems that the harvesting site and the level of negative pressure do not have a crucial or limiting effect on basic ADSC characteristics.*in vitro* culturing and for use in tissue engineering, it seems that the harvesting site and the level of negative pressure do not have a crucial or limiting effect on basic ADSC characteristics.

## 1. Background

Stem cells of various origin are fundamental elements for cell-based therapies in regenerative medicine, particularly for tissue engineering. Nowadays, tissue engineering tends to use stem cells that (1) are pluripotent or multipotent, (2) can be routinely harvested in large quantities, and (3) are surrounded by fewer ethical issues than other types. Mesenchymal stromal cells (MSCs) are multipotent plastic-adherent fibroblast-like cells. They can be harvested predominantly from adult organs and tissues, i.e., bone marrow, peripheral blood, adipose tissue, skin, skeletal muscle, dental pulp, brain, and endometrium [1]. Not only adult tissues but also extrafoetal tissues, such as placenta, umbilical cord tissue, amniotic membrane, and amniotic fluid can also serve as sources of MSCs. The characteristics and the differentiation of bone marrow-derived stromal cells (BMSCs) have been widely studied, as they were the first MSCs to be described. BMSCs provide favourable differentiation characteristics. However, the BMSC harvesting procedure is uncomfortable for donors and adipose tissue-derived stromal cells (ADSCs) provide similar yields of isolated cells, together with greater subsequent proliferation capacity [2]. In recent years, ADSCs have become an ideal target for tissue engineering and cell-based therapies. A relatively easy harvesting procedure and the multipotent characteristics of ADSCs make these stromal cells suitable for various uses [3]. The possibility of autologous application in cell-based therapies can be a further advantage of ADSCs.

The methods for isolating ADSCs from adipose tissue can be divided into enzymatic and nonenzymatic approaches [4, 5]. Until now, enzymatic digestion using collagenase has been the most widely performed procedure. However, newer alternative nonenzymatic techniques (e.g., vibration and centrifuging) can also be applied, especially for clinical purposes [6]. After enzymatic digestion and centrifugation, three separated parts are obtained, namely, the upper oily part containing adipocytes, the middle part consisting of digested tissue, and the reddish stromal vascular fraction (SVF) pellet at the bottom [7]. The SVF part is a mixture of distinct cell types consisting of ADSCs and variably also of pericytes, preadipocytes, endothelial precursor cells, endothelial cells, macrophages, smooth muscle cells, fibroblasts, and lymphocytes [5].

A large number and range of studies focused on obtaining ADSCs have been published. The studies have investigated various fat-harvesting procedures, cell isolation procedures, and donor factors. All these factors can influence the viability, the yields, and the subsequent proliferation and differentiation of the isolated cells. Tumescent liposuction is used as one of the easiest procedures for harvesting adipose tissue. The negative pressure (vacuum) that is used during the liposuction procedure is an important factor that influences the quality and the amount of harvested tissue. Lee et al. studied the effect of different negative pressures (i.e., -381 mmHg and -635 mmHg) on fat grafting [8]. In their *in vivo* study, no significant differences in the weight or in the histology of the fat grafts were observed; moreover, higher negative pressure did not affect the viability of the fat grafts [8]. Similarly, in a study by Charles-de-Sá et al., no significant differences, either in the viability of the adipocytes or in the number of MSCs, were found in adipose tissue obtained under various negative pressures [9]. However, other studies have reported a significant influence of negative pressure on cell characteristics. Mojallal et al. measured greater cell yields in adipose tissue harvested under a lower negative pressure (-350 mmHg) than under a higher negative pressure (-700 mmHg) [10]. Similarly, Chen et al. reported more than 2-fold higher cell numbers in SVF isolated from adipose tissue harvested under a lower negative pressure (−225 mmHg ± 37 mmHg) than under a higher negative pressure (−410 mmHg ± 37 mmHg) [11]. They also reported faster cell growth and higher secretion of some growth factors in cells obtained under lower negative pressure in the initial passages [11].

The harvesting site of the superficial adipose tissue seems to be another important donor factor potentially influencing the viability and the proliferation of the isolated cells. Jurgens et al. compared the numbers of cells isolated from the abdomen area and from the hip/thigh area. They found a significantly higher frequency of ADSCs in SVF isolates derived from the abdomen area, but no significant differences were found in the absolute numbers of nucleated cells [12]. However, the osteogenic and chondrogenic differentiation capacity of the ADSCs was not affected by the harvesting site [12]. Padoin et al. observed higher cell yields from the lower abdomen and from the inner thigh than from other liposuction areas (i.e., upper abdomen, flank, trochanteric area, and knee) [13]. Differences in the viability and in the amount of SVF and in the numbers of ADSCs after culturing, were also studied by Tsekouras and coworkers. In their study, the SVF from the outer thigh exhibited higher cell numbers [14]. This tendency also continued in subsequent cell culturing, where the outer and inner thigh samples both showed higher numbers of ADSCs than the abdomen, waist, or inner knee samples. Other studies reported no statistically significant differences in the volumes of fat grafts [15, 16] or in adipocyte viability [17] according to the donor sites.

Not only the negative pressure during liposuction and in the donor harvesting site but also different harvesting procedures [18] and other individual donor factors have been found to influence the viability, proliferation, and differentiation characteristics of ADSCs. Further factors include body mass index (BMI), age, gender, intercurrent diseases, such as diabetes mellitus, and also radiotherapy and drug treatment [19].

There is a need to investigate and confirm the best harvesting conditions for ADSCs, which could help to bring them into routine use in clinical practice. Until now, studies have not been uniform and have been focused predominantly on different cell types (adipocytes, preadipocytes, total SVF). The potential differences in the characteristics of ADSCs seem to be nonnegligible and need to be further clarified for future use in tissue engineering. The objective of our study was to investigate the influence of negative pressure during liposuction and also of the donor site on the yields of initially attached cells and on subsequent cell proliferation, achieved cell numbers, cell viability, diameter, and phenotypic markers of isolated ADSCs when cultured in *in vitro* conditions.

## 2. Materials and Methods

### 2.1. Group of Donors and Liposuction Procedure

**Scheme 1:**
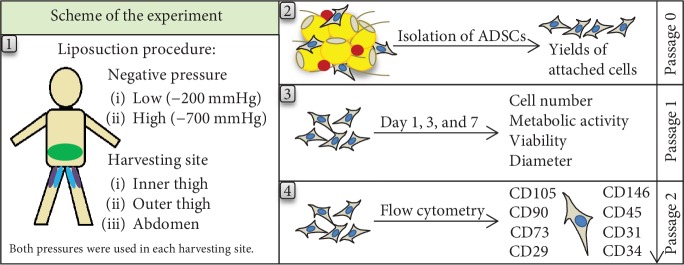
Scheme of the experiment. Sites in the abdomen, the inner thigh, and the outer thigh where liposuction at low negative pressure (-200 mmHg) and at high negative pressure (-700 mmHg) was performed. After the cell isolation, the initial yields of attached cells were counted. In subsequent passages, the number, viability, diameter, doubling time, mitochondrial activity (all in passage 1), and CD surface markers (passage 2) of isolated ADSCs were evaluated.

A comparative study was performed on samples of subcutaneous adipose tissue from 15 healthy donors after informed consent at Hospital Na Bulovce in Prague. The group of females (*n* = 14) and one male (*n* = 1) underwent tumescent liposuction, whereby adipose tissue from the inner thigh (*n* = 3), from the outer thigh (*n* = 7), and from the abdomen (*n* = 9) was harvested. Harvesting was conducted in compliance with the tenets of the Declaration of Helsinki on experiments involving human tissues and under ethical approval issued by the Ethics Committee of Hospital Na Bulovce in Prague (August 21, 2014). The liposuctions were performed under sterile conditions, using tumescence. The tumescent solution contained a 1000 mL of physiological solution with adrenaline (1 : 200,000) 1 mL and bicarbonate 8.4% 20 mL. In order to protect the harvested stromal cells from possible toxicity, no local anaesthetics were used. We used a liposuction machine (MEDELA dominant) that enabled continuous negative pressure to be set, and we utilized negative pressure of -200 mmHg and -700 mmHg. Superficial fat tissue was harvested using a Coleman Style blunt cannula with 4 holes and an inner diameter of 3 mm. Both low negative pressure (i.e., -200 mmHg) and high negative pressure (i.e., -700 mmHg) were used during liposuction in selected harvesting sites for each donor. Specifically, in the abdominal region, low pressure was used on one side of the abdomen, while high pressure was applied on the opposite side of the abdomen. Similarly, in the outer and inner thigh regions, low pressure was applied on one leg and the high pressure was applied on the contralateral leg (Scheme 1). A different cannula and vacuum suction container was used for low and high pressure harvesting to prevent contamination of low pressure harvesting material with high pressure harvesting material and vice versa. The age range of the donors was 26–53 years (mean age 37.8 ± 7.8 years) and the BMI range was 19.60–36.17 kg/m^2^ (mean BMI 25.44 ± 4.37 kg/m^2^) (Table 1). The donors did not suffer from diabetes or from hypertension, and they were not tobacco users.

### 2.2. Isolation of ADSCs

The isolation procedure was performed in fresh lipoaspirates (within 2 hours after the liposuction procedure) according to the isolation protocol by Estes et al. [7]. However, we made some slight modifications, as described in our previous study [20]. In brief, the lipoaspirates were washed several times with phosphate-buffered saline (PBS; Sigma-Aldrich). Then, the lipoaspirate was digested, using PBS containing 1% (wt/vol) bovine serum albumin (BSA; Sigma-Aldrich) and type I collagenase 0.1% (wt/vol) (Worthington) for 1 hour at a temperature of 37°C. After the digestion procedure, the tissue was centrifuged, and the upper and middle layers were aspirated. The obtained SVF was washed three times. A filter with pores 100 *μ*m in size (Cell Strainer, BD Falcon) was additionally used to filter the cell suspension of SVF right before seeding into culture flasks (75 cm^2^, TPP, Switzerland) in a density of 0.16 mL of original lipoaspirate/cm^2^. The isolated cells were cultured in Dulbecco's modified Eagle medium (DMEM; Gibco), supplemented with 10% (vol/vol) foetal bovine serum (FBS; Gibco), gentamicin (40 *μ*g/mL; LEK), and recombinant human fibroblast growth factor basic (FGF2; 10 ng/mL; GenScript). The primary cells, referred to as “passage 0,” were cultured until they reached 70%–80% confluence. Then, the cells were passaged.

For the experiments that followed (Scheme 1), the cells isolated from the lipoaspirate harvested under low negative pressure (i.e., -200 mmHg) are referred to as “low,” and the cells isolated from the lipoaspirate harvested under high negative pressure (i.e., -700 mmHg) are referred to as “high.” The compared groups of cells are referred to as low inner thigh (low I thigh), high inner thigh (high I thigh), low outer thigh (low O thigh), high outer thigh (high O thigh), low abdomen, and high abdomen.

### 2.3. Yields of Initially Attached Cells

For the primary culture of isolated cells, as mentioned above, the seeding density was 0.16 mL of original lipoaspirate/cm^2^. On day 1 after isolation and seeding (passage 0), the culture medium was changed with the fresh medium, and the unattached cells were washed away. Then, the cell yields per 1 mL of lipoaspirate were counted from the number of attached cells, because only these cells are relevant for potential use in tissue engineering. Microphotographs of 4 to 6 randomly chosen microscopic fields for each sample were taken by phase-contrast microscope and were analysed by manual cell counting. Then, the number of attached cells was compared depending on different negative pressure or on the harvesting site.

### 2.4. Cell Number, Viability, Diameter, and Doubling Time

The cells from each donor, harvested under low and high negative pressure within the corresponding areas in the abdomen or in the thigh, were cultured and then analysed. The isolated cells in passage 1 were seeded into 12-well tissue culture polystyrene plates (TPP, Switzerland; well diameter 2.1 cm) in a density of 14,000 cells/cm^2^ (i.e., 50,000 cells/well) and were cultivated in DMEM+10% (vol/vol) FBS+10 ng/mL FGF2 for 7 days. The volume of the cell culture medium was 3 mL/well. The cells were cultivated in a humidified air atmosphere with 5% CO_2_ at a temperature of 37°C. On days 1, 3, and 7, the cells were washed with PBS and were then detached by incubation with Trypsin-EDTA Solution (Sigma-Aldrich) for 4 minutes at 37°C. The effect of the Trypsin-EDTA solution was subsequently inhibited by adding a medium with FBS, and the cells were resuspended. The number, the viability, and the diameter of the detached cells in each well were measured using a Vi-CELL XR Cell Viability Analyzer (Beckman Coulter). In this analyser, the cell viability is evaluated by a trypan blue exclusion test. From 5 to 8 independent samples for each experimental group of a donor in each time interval were analysed. The cell population doubling time (DT) was calculated from the ADSC numbers, according to the following equation: DT = *t* × ln(2)/(ln(*N*)–ln(*N*_0_)), where *t* represents the duration of culture, *N* represents the number of cells on day 3, and *N*_0_ represents the number of cells on day 1.

### 2.5. Cell Mitochondrial Activity

The activity of mitochondrial enzymes is generally measured in order to estimate the cell proliferation activity. The isolated cells in passage 1 were seeded into 24-well tissue culture polystyrene plates (TPP, Switzerland; well diameter 1.5 cm) in a density of 14,000 cells/cm^2^ (i.e., 25,000 cells/well) and were cultivated in DMEM+10% FBS+10 ng/mL FGF2 for 7 days. The volume of cell culture medium was 1.5 mL/well. On days 3 and 7, a CellTiter 96® Aqueous One Solution Cell Proliferation Assay (MTS; Promega Corporation) was performed according to the manufacturer's protocol. In brief, the principle of the MTS assay is based on a colorimetric change of the yellow tetrazolium salt to brown formazan. This change is brought about by the activity of mitochondrial enzymes. The absorbance was measured at a wavelength of 490 nm, using a VersaMax ELISA microplate reader (Molecular Devices LLC). From 5 to 6 independent samples were measured for each experimental group in each time interval.

### 2.6. Flow Cytometry

In passage 2, the cells were characterised by flow cytometry, using antibodies against specific surface CD markers. An evaluation was made of the percentage of cells in the population that contained standard markers of ADSCs, i.e., CD105 (also referred to as endoglin, a membrane glycoprotein which is part of the TGF-*β* receptor complex), CD90 (Thy-1, a thymocyte antigen belonging to the immunoglobulin superfamily), and CD73 (ecto-5′-nucleotidase, a glycosylphosphatidylinositol-anchored membrane protein). Other evaluated markers included CD29 (integrin *β*_1_, a component of receptors for collagen and fibronectin), CD146 (a melanoma cell adhesion molecule, a receptor for laminin), CD31 (also referred to as platelet-endothelial cell adhesion molecule-1, PECAM-1), and hematopoietic cell markers CD34 and CD45 [3]. In brief, the cells were washed with PBS and were incubated with Trypsin-EDTA for 4 minutes at 37°C. Subsequently, the medium with FBS was added and the cells were centrifuged (5 min, 300 g). The supernatant was aspired off, and the cells were resuspended in PBS with 0.5% (wt/vol) BSA (Sigma-Aldrich). The cells were equally divided into aliquots (i.e., 250,000 cells/aliquot). FITC-, Alexa488-, Alexa647-, or PE-conjugated monoclonal antibodies, i.e., against CD105, CD45 (Exbio Praha), CD90 (BD Biosciences), CD73, CD146, CD31 (BioLegend), CD29 and CD34 (Invitrogen), were added separately into aliquots. The aliquots were incubated with the antibodies for 30 minutes at 4°C in dark conditions. Next, the stained cells were washed three times with PBS with 0.5% (wt/vol) BSA and were analysed with the Accuri C6 Flow Cytometer System (BD Biosciences). In each aliquot, 20,000 events were recorded for each CD surface marker.

### 2.7. Microscopy Techniques

Phase-contrast microscopy was used to visualise the process of attachment, spreading, and growth in native ADSCs after isolation (passage 0). The immunofluorescence staining of CD surface markers was performed on native adhering ADSCs (passage 2) using PE-CD90 (BD Science) and Alexa488-CD29 (Invitrogen) antibodies. Cell nuclei in native cells were counterstained with Hoechst 33342 (Sigma-Aldrich) for 30 minutes at room temperature in the dark. Olympus microscope IX71 (objective magnification 10x or 20x) was used to take representative images.

### 2.8. Statistical Analysis

First, to evaluate the significance of different negative pressures, the observed data (i.e., initial cell yields, later cell numbers, and mitochondrial activity) were presented as the ratio of low-pressure cells to high-pressure cells for each donor. The Mann-Whitney Rank Sum test was used to test the equality of the medians of the ratios on different days of the experiment. Second, an unpaired two-sample *t*-test (for parametric data) or a Mann-Whitney Rank Sum test (for nonparametric data) was used to test the significance of the differences between the outer thigh area and the abdomen area. The inner thigh region was not statistically compared with other harvesting sites due to a relatively small group of samples (i.e., from only 3 patients). All the measured data were tested for normality according to the Kolmogorov-Smirnov test. Data which showed a Gaussian distribution are expressed as mean ± SD. However, due to the small sample size and the wide dispersion among the donors, some of the data did not show a Gaussian distribution. The nonparametric data are expressed as the median and the interquartile range (IQ range). The statistical analysis was performed using SigmaStat Software (Systat Software Inc., USA); *p* < 0.001 (for flow cytometry) or *p* < 0.05 (for all other methods) was considered statistically significant. The plots were generated in R (programming language).

## 3. Results

### 3.1. Growth of Cells after Isolation and Cell Yields

**Figure 1:**
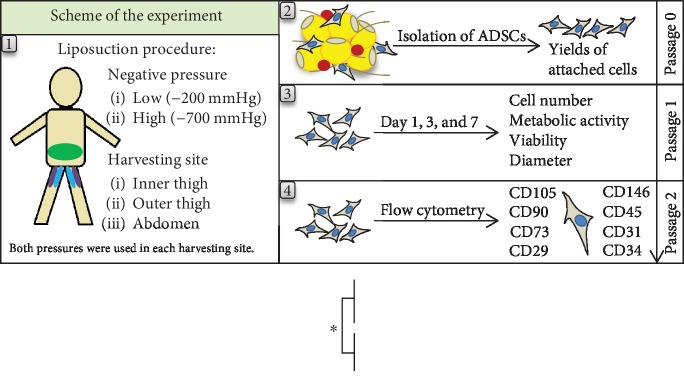
The process of attachment, spreading, and growth in ADSCs from the same patient on days 2, 5, and 7 after isolation. The ADSCs were isolated from the inner thigh area and from the abdomen area, under low negative pressure (-200 mmHg) and under high negative pressure (-700 mmHg). Passage 0. Scale bar 200 *μ*m. Representative images are shown.

**Figure 2:**
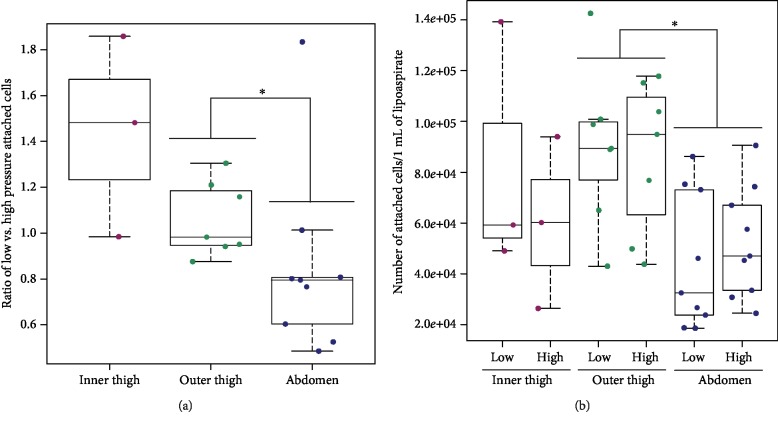
Cell yields counted from the number of initially attached cells. (a) The ratio of the number of low-pressure cells to the number of high-pressure cells for each donor on day 1 after isolation; passage 0. *p* < 0.05 (^∗^) is for harvesting area (outer thigh vs. abdomen) significance testing. (b) The number of attached cells per 1 mL of lipoaspirate; passage 0. *p* < 0.05 (^∗^) is for harvesting area significance testing (outer thigh vs. abdomen). The inner thigh region was not statistically compared to the outer thigh and abdomen regions due to the relatively small sample size.

In passage 0, we observed slight differences in the range of cell adhesion and growth among the cells harvested from various donors. However, the cells from all donors usually reached 70% or 80% confluence by day 10.  Figure 1 shows representative images of the process of adhesion and growth in ADSCs after isolation from the same patient. On day 1 after isolation, the number of attached cells per 1 mL of lipoaspirate was counted in each sample. The ratio of attached low-pressure cells to attached high-pressure cells for each donor showed a median level near to 1.0 for the outer thigh region, which means a similar number of attached cells for both pressures (Figure 2(a)). However, the median level of this ratio (0.79) was significantly lower for the abdomen region (Figure 2(a)) which indicates higher cell yields from high-pressure lipoaspirates from this harvesting site. We observed a significantly 2-fold or 3-fold higher number of attached cells from the outer thigh region than from the abdomen region (Figure 2(b)). The inner thigh region was not statistically compared with other harvesting sites due to the relatively small group of samples.

### 3.2. Cell Number

**Figure 3:**
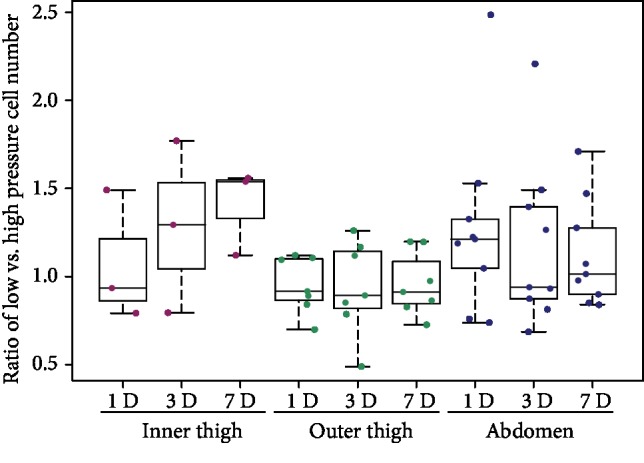
The influence of negative pressure on the number of ADSCs. The ratio of the number of low-pressure cells to the number of high-pressure cells for each donor. The measurements were performed on ADSCs from the inner thigh (*n* = 3) area, from the outer thigh (*n* = 7) area, and from the abdomen (*n* = 9) area on day 1 (1D), day 3 (3D), and day 7 (7D); passage 2. No significant differences among the groups were observed.

**Figure 4:**
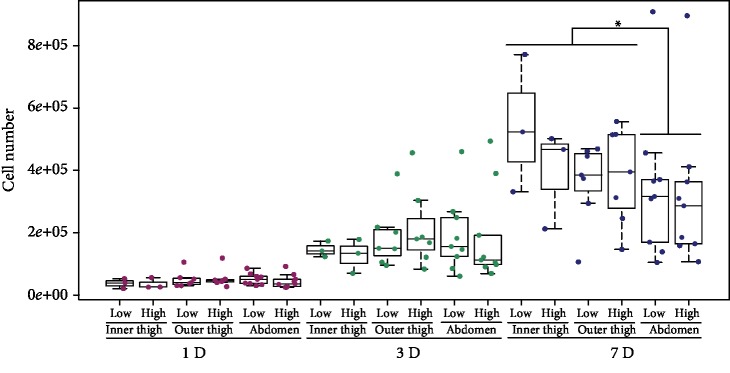
The number of ADSCs. The ADSCs were harvested under low pressure and under high pressure from the inner thigh area, the outer thigh area, and the abdomen area. Days 1, 3, and 7; passage 2. On day 7, the thigh ADSCs (i.e., inner thigh+outer thigh) reached significantly higher (*p* = 0.048) cell numbers than the abdomen ADSCs. *p* < 0.05 (^∗^) is for harvesting area significance testing.

The number of cells obtained from the corresponding areas of the abdomen or the thigh under low negative pressure and under high negative pressure for the same donor was measured on days 1, 3, and 7. The ratio of the number of low-pressure cells to the number of high-pressure cells on a specific day of the culture from each donor showed median levels near to 1.0 in cells from the inner thigh, outer thigh, and abdomen areas (Figure 3). There were no statistical differences in cell numbers between the outer thigh and abdomen areas on days 1 and 3 (Figure 4). When the groups of cells from the inner thigh and the outer thigh were evaluated together, we observed higher cell number in thigh ADSCs than in abdomen ADSCs (*p* = 0.048) on day 7 (Figure 4).

### 3.3. Doubling Time

**Figure 5:**
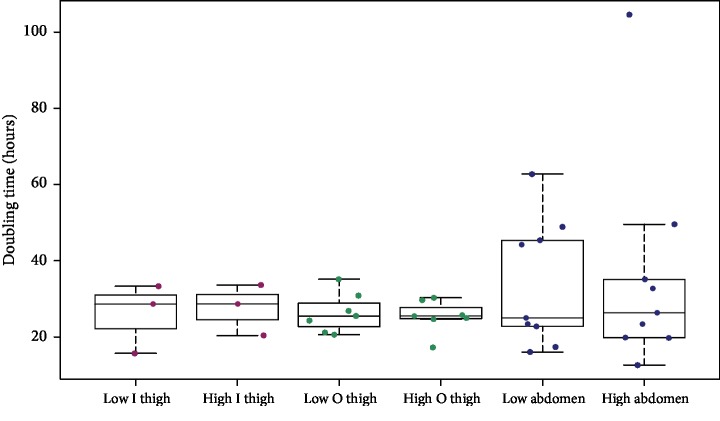
Population doubling time. Population doubling time of low-pressure ADSCs and high-pressure ADSCs from the inner thigh (*n* = 3) area, from the outer thigh (*n* = 7) area, and from the abdomen (*n* = 9) area. No significant differences were observed among the groups investigated here.

The doubling time was calculated between days 1 and 3 (i.e., 48 hours of cell culture). There were similar median values in all sample groups, from 24.99 hours (low abdomen) to 28.65 hours (high inner thigh) (Figure 5). No significant differences were observed between the sample groups.

### 3.4. Viability and Diameter

No significant differences were found in the viability of the cells, measured by the trypan blue exclusion test, on day 1 (from 88.0% for low abdomen to 93.6% for low outer thigh), on day 3 (from 93.5% for high abdomen to 96.6% for high outer thigh), and on day 7 (from 90.3% for high inner thigh to 95.9% for high outer thigh) (Table 2). We observed significantly larger diameter of outer thigh ADSCs than of abdomen ADSCs (*p* = 0.038) on day 1. However, no significant differences in diameter were observed on day 3 and on day 7 (Table 3).

### 3.5. Cell Mitochondrial Activity

**Figure 6:**
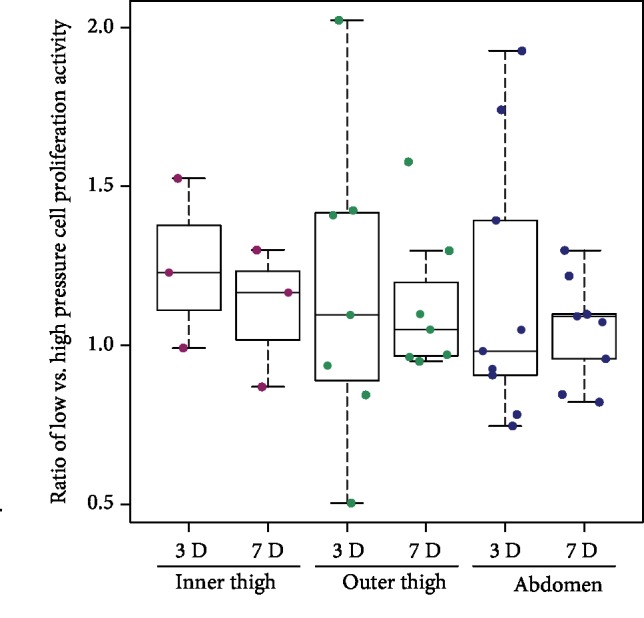
The influence of negative pressure on the mitochondrial activity of ADSCs. The ratio of the mitochondrial activity of low-pressure cells to the mitochondrial activity of high-pressure cells obtained for each donor. The measurements were performed on ADSCs from the inner thigh (*n* = 3) area, from the outer thigh (*n* = 7) area, and from the abdomen (*n* = 9) area on day 3 (3D) and on day 7 (7D). No significant differences among the observed groups were observed.

The activity of mitochondrial enzymes in ADSCs, considered as an indirect indicator of cell proliferation activity, was measured on days 3 and 7 after seeding. The ratio of the mitochondrial activity of the low-pressure cells to the mitochondrial activity of the high-pressure cells on a specific day of the culture from each donor revealed median levels near to 1.0 in cells from the inner thigh, outer thigh, and abdomen areas, and no significant differences were observed between the low-pressure cells and the high-pressure cells (Figure 6). Similarly, there were no significant differences in the mitochondrial activity of cells from different donor sites on day 3 (Table 4). On day 7, we observed a tendency toward lower mitochondrial activity of inner thigh ADSCs than of other harvesting sites; however, no statistical analysis was performed due to the relatively small sample size.

### 3.6. Flow Cytometry

**Figure 7:**
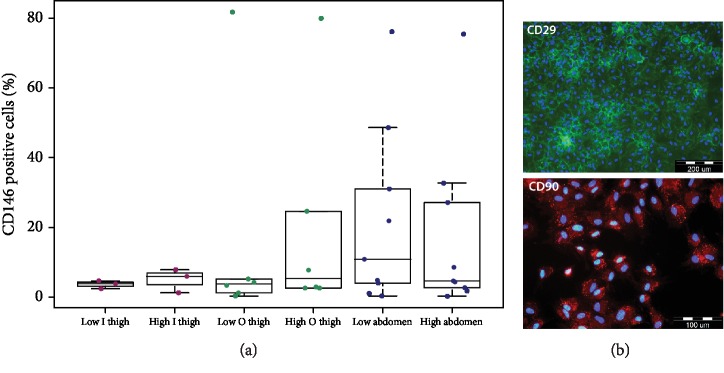
(a) The percentage of CD146-positive cells in each group of cells. No significant differences among the harvesting sites were observed. (b) The immunofluorescence staining of CD29 and CD90 in ADSCs. Cell nuclei are counterstained with Hoechst 33342. Olympus microscope IX71. Scale bar 200 *μ*m (CD29) and 100 *μ*m (CD90).

The percentage of cells positive for typical markers of mesenchymal stromal cells, i.e., CD105, CD90, CD73, and CD29, was very high in ADSCs obtained from all tested sources. No significant differences were found in the presence of these markers in cells obtained from lipoaspirates taken at different negative pressures and from different harvesting sites (Table 5). However, slightly lower and more variable values were obtained in abdomen-derived ADSCs. Representative images of CD90 and CD29 immunostaining are shown in Figure 7(b). We also observed variability in the percentage of CD146^+^ cells among the donors (from 3.9% in low inner thigh and low outer thigh to 10.9% in low abdomen) (Figure 7(a)). This variability was slightly higher in ADSCs from the abdomen area and was not dependent on negative pressure. The percentage of cells bearing hematopoietic and endothelial cell markers, namely, CD45, CD34, and CD31, was very low and showed no significant differences between cells obtained at different negative pressures and from different donor sites (Table 5).

## 4. Discussion

A set of experiments was performed to reveal the influence of negative pressure and harvesting site on the characteristics of isolated ADSCs from a number of donors. For future use in tissue engineering, we were mainly interested in significant differences in the basic adhesion and growth characteristics of ADSCs in passages 1 and 2 after isolation. Our study provided an opportunity to compare isolated cells from the same topographic area that had been harvested under low negative pressure and under high negative pressure from each donor. In passage 0, we observed slight differences in the rate of attachment and spreading and in the growth of the ADSCs of the donors after the cells had been isolated. These initial interdonor differences may have been caused by differences in ADSC frequency in the obtained SVF cells. Varying frequencies of ADSCs, determined by a colony-forming unit assay and/or by a limiting dilution assay, have been found in the adipose tissue harvested from various donor sites [12] or when different harvesting procedures are used [21]. Specifically, Jurgens et al. observed significantly higher frequency of ADSCs isolated from adipose tissue harvested from the abdomen region than from the hip/thigh region [12]. Oedayrajsingh-Varma et al. observed a significantly higher frequency of ADSCs isolated from adipose tissue obtained by resection and tumescent liposuction than from tissue obtained by ultrasound-assisted liposuction [21]. In those studies, the absolute number of nucleated cells in the harvested adipose tissue and the number of viable cells in the stromal vascular fraction were not affected by the anatomical site or by the type of surgical procedure. However, in other studies, the anatomical site did have an influence on the total SVF and on the ADSC yields. Iyyanki et al. observed significantly higher total SVF yields from the abdominal harvesting site than from the flank and axilla harvesting sites; however, the ADSC yields did not differ significantly [18]. In a study by Fraser et al., the abdomen-adipocyte yield was 1.7-fold higher than the hip-adipocyte yield, and the adipocyte yields displayed large donor-to-donor variabilities [22]. However, neither the nucleated cell yields nor the preadipocyte yields differed significantly [22]. A large range of ADSC yields among donors was also observed, and no statistical differences were found between the abdomen, the thigh, and the mammary areas [21]. By contrast, our study showed a potential influence of harvesting site, as we observed a higher number of attached cells per 1 mL of lipoaspirate for the outer thigh area than for the abdomen area on day 1 after isolation in *in vitro* culture. Different results concerning the influence of harvesting site on cell yields might be obtained because of the differences in the target cell populations being studied in different papers. For plastic surgery purposes, the cell yields of all nucleated cells, adipocytes, preadipocytes, and SVF are also a subject of interest. However, tissue engineering focuses more on the yields of adherent ADSCs that can be further proliferated and/or differentiated.

The total number of harvested cells can also be influenced by the level of negative pressure used during the liposuction procedure. In a study by Mojallal et al., a lower negative pressure (-350 mmHg) during liposuction resulted in higher SVF yields than a higher negative pressure (-700 mmHg) [10]. Similarly, in a more recent study by Cheriyan et al., higher counts and higher viability of adipocytes were found in lipoaspirates obtained at a lower negative pressure (-250 mmHg) than at a higher negative pressure (-760 mmHg) [23]. However, each of these studies was performed on three patients only. In our study, the number of attached cells after the isolation was similar for low- and high-pressure cells from the outer thigh region, whereas the abdomen region was characterised by initial higher cell yields of attached cells for high pressure.

Although the initial SVF yields, adipocyte yields, and ADSC frequency in lipoaspirates can vary, later differences during *in vitro* ADSC culturing were of particular interest to us. Our study was focused on the number, the mitochondrial activity, and the viability of the ADSCs in subsequent passaging. We observed similar cell numbers and mitochondrial activity independently of low- and high-negative pressure for a specific region. This means that the subsequent proliferation of ADSCs was not affected by the negative pressure used during the liposuction procedure. Chen et al. observed initial higher proliferation activity (assessed by Cell Counting Kit-8) in lower negative pressure SVF cells than in higher negative pressure SVF cells from the abdominal area in passages 1 and 2 [11]. However, these significant differences did not appear in passage 3 [11]. Similarly, our results could also provide support for the theory that the differences in proliferation activity between low-pressure cells and high-pressure cells become less noticeable after passaging during *in vitro* cultivation. Interestingly, other researchers have reported that different apparatuses and different levels of negative pressure during liposuction do not influence the percentage and the viability of adipocytes and isolated mesenchymal stromal cells [9]. The discrepancies among the comparative studies may also have arisen because different cell populations were being studied. That is, negative pressure techniques may have a bigger effect on adipocytes, due to their bigger size, while they may have only a minimal effect on smaller cells, including progenitor cells [22]. It is therefore necessary to consider carefully which types of cells from adipose tissue are to be harvested and used. In our study, the outer thigh ADSCs were bigger in diameter in the cell suspension on day 1 after seeding than the abdomen ADSCs. However, the cells were of similar diameters on days 3 and 7.

The function and the representation of cell types in adipose tissue vary among the topographic regions. Preadipocytes and ADSCs obtained from subcutaneous, mesenteric, omental, or intrathoracic fat depots display distinct expression profiles and differentiation capacity [24, 25]. Subcutaneous fat depots are easier to obtain than other fat depots. Although the morphology of subcutaneous and visceral fat did not differ significantly, the harvested subcutaneous ADSCs displayed significantly higher cell numbers, a shorter doubling time, and higher CD146 expression than for visceral ADSCs in later passages [26]. Moreover, within the subcutaneous depots, superficial depots seem to have better stemness and multipotency characteristics of the cells than deep subcutaneous depots [27]. Until now, the harvesting site of fat depots has usually been selected on the basis of actual need or choice. However, the particular anatomic source of adipose tissue harvesting can play a role in further reconstructive surgery and cell-based therapies. The cells from different fat depots express different homeobox (Hox) genes. This supports the idea that they are of different embryonic origin, and so the donor and the host adipose tissue sites need to be carefully matched [28]. Kouidhi et al. compared the gene expression of human knee ADSCs with chin ADSCs [29]. They found more enhanced expression of Pax3 (i.e., a neural crest marker) in chin ADSCs than in knee ADSCs, whereas the expression of most of the Hox genes that are typical for the mesodermal environment was higher in knee ADSCs than in chin ADSCs. In later passages, chin ADSCs also displayed higher self-renewal potential [29]. In our study, we obtained similar numbers and similar viability of ADSCs from the inner thigh area, the outer thigh area, and the abdomen area on days 1 and 3. Thus, our results are in accordance with studies by other researchers, in which similar growth kinetics were found in ADSCs from the abdomen area and from the hip/thigh area [12, 30]. However, with similar cell numbers on days 1 and 3, we observed a tendency of thigh ADSCs (inner thigh+outer thigh) to reach higher values than abdomen ADSCs on day 7 (*p* = 0.048). It therefore seems that there may be a significant difference in later cell numbers between the harvesting sites for most of the patients included in our study, though we observed large variation among the donors. Interestingly, we also observed a tendency toward lower mitochondrial activity of inner thigh ADSCs than of outer thigh ADSCs and abdomen ADSCs on day 7. These results may correspond with the slightly higher cell numbers of inner thigh ADSCs on day 7, when the cells have already reached confluence and have reduced their proliferation activity. However, the smaller number of inner thigh ADSC samples than in the case of other groups (i.e., outer thigh ADSCs and abdomen ADSC) may also have affected the results. The harvesting site can also influence the colony-forming unit (CFU) in isolated ADSCs. Fraser et al. observed that the CFU was higher in hip ADSCs than in abdomen ADSCs [22]. This finding could be in accordance with a higher proliferation rate of hip/thigh ADSCs in later time intervals of the culture [22].

During our experiments, we observed a nonparametric distribution of the donors' data. The interdonor variabilities that were not dependent on the harvesting site or on negative pressure may have been caused by other donor factors. Age and BMI are other factors known to play a considerable role in SVF and ADSC yields and characteristics [19]. However, research findings regarding the influence of age and BMI on ADSC yields are often contradictory [31–33]. For example, in the study by de Girolamo et al., the cellular yield of ADSCs was significantly greater from older patients than from younger patients [31], while in the study by Faustini et al., the patient's age seemed not to influence the cell yield [32]. Significant donor-to-donor variability has also been reported in multilineage differentiation capacity, self-renewal capacity, and immunomodulatory cytokine secretion [34]. Although some of these variabilities can be explained by a medical history of breast cancer and subsequent treatment, there were also significant differences among donors who had not been diagnosed with cancer [34]. Atherosclerosis is another donor factor which can alter the secretome and reduce the immunomodulatory capacity of ADSCs due to impaired mitochondrial functions [35]. In addition, the ADSCs isolated from patients with renovascular disease exhibited a higher level of DNA damage and lower migratory capacity than ADSCs from healthy donors [36]. In another study, ADSCs isolated from patients suffering from scleroderma, an autoimmune connective tissue disease, showed a lower proliferation rate and lower migration capacity than in the control ADSCs from healthy donors [37].

Many papers have reported on various donor-to-donor factors that have a potential impact on the characteristics of mesenchymal stromal cells. In addition, it seems that there are many cell-to-cell variations within the same donor. This cell-to-cell heterogeneity can be manifested both *in vitro* and *in vivo* by interclonal functional and molecular variation, e.g., variable differentiation capacity, existing fast-growing and slow-growing clones, and other differences in proteome and transcriptome [38]. The percentage of various clones in MSCs develops and changes during cell passaging. Even within a single MSC clone, there is a growing body of evidence that the intraclonal heterogeneity alters cell behaviour and characteristics [38].

In most of the donors, we proved a high level of positivity of the isolated cells for CD105, CD90, CD73, and CD29 (>80% in ADSCs) and a low level of positivity or absence of CD45, CD31, and CD33 (≤2% in ADSCs), according to the guidelines for characterizing ADSCs [39]. We observed no significant differences in the presence of CD markers depending on negative pressure or on harvesting site. Our results are in accordance with those reported by other researchers, who have found no differences in CD markers in SVF harvested from different sites [12, 14, 30]. In another study, the presence of pericytes, progenitor endothelial cells, preadipocyte cells, and mesenchymal cells in SVF was not influenced by different negative pressures [9]. In addition, in the study by Chen et al., where higher negative pressure had a negative influence on yields, on growth, and on the secretion of growth factors, no differences in CD markers were found [11]. Interestingly, we observed variability in the presence of CD146 among the donors. The presence of CD146^+^ cells in subcutaneous depots was also not negligible in a study by Lee et al. [26]. CD146 positivity can be a sign of pericytes. Pericytes are cells in contact with small vessels in the adipose tissue, and they are also present in the harvested SVF [40]. The origin of the pericyte is not the only possible explanation. For a review of other theories explaining the presence of CD146, see [41]. In MSCs, high expression of CD146 is associated with a commitment towards vascular smooth muscle cell lineage [42]. This commitment could be interesting for vascular tissue engineering, when differentiating ADSCs towards vascular smooth muscle cells is required. CD146^+^ cells in combination with human umbilical vein endothelial cells (HUVECs) were also reported to support the formation and the elongation of capillary-like tubular structures [26]. Lee et al. also observed greater proliferation of CD146^+^ cells than of CD146^−^ cells; however, the percentage of CD146^+^ cells in an ADSC culture decreased with subsequent subculturing [26]. It seems that the CD146 expression among ADSCs is relatively heterogeneous and could play an important role in potential specific tissue engineering applications. The presence of other hematopoietic and endothelial cell markers (e.g., CD34, CD45, and CD31) can influence future therapies using SVF or ADSCs. The optimal ratio of ADSCs and hematopoietic stem cell progenitors in isolated SVF defined by specific CD surface markers seems to be the key for successful stem cell therapies [43].

### 4.1. Limitation

The first limitation of our study is the relatively small sample size, with uneven numbers of samples from each donor site (i.e., inner thigh (*n* = 3), outer thigh (*n* = 7), and abdomen (*n* = 9)). Due to the smallest sample size of inner thigh ADSCs, we did not make a statistical comparison between this group of cells and outer thigh ADSCs or abdomen ADSCs. A greater number of donors would be desirable. However, we assume that for ADSC characterization under *in vitro* culture conditions and for later tissue engineering purposes, the sample size is sufficient.

The second limitation of the study is that it was primarily focused on negative pressure and on the harvesting site and not on other patient factors, such as age, gender, or BMI; these other characteristics were therefore not completely uniform among the donors. Nevertheless, the studied groups showed similar age and BMI parameters with normal data distribution.

The third limitation of the study is that it was focused on the later use of ADSCs in tissue engineering. Therefore, we characterized only the fraction of isolated ADSCs that adhered to the plastic culture flasks. The yields of ADSCs were counted after they had adhered to the flasks, and their characteristics (cell proliferation, flow cytometry analysis of surface markers) were studied in subsequent passages. No other cell types (i.e., adipocytes or all nucleated cells) were analysed in this study with respect to their yields or their viability. The conclusions concerning the influence of negative pressure and harvesting site therefore refer only to plastic-adherent ADSCs.

To characterize the ADSCs in *in vitro* culture conditions, we chose passage 1 and passage 2 depending on specific analyses. These passages were the same for all analysed ADSCs. However, the growth dynamics of the cells is known to vary from passage to passage, and this variability can also be specific in each isolated ADSC population.

## 5. Conclusion

In our study, we observed a significantly higher number of initially attached cells per 1 mL of lipoaspirate for the outer thigh region than for the abdomen region on day 1 after isolation. Different negative pressure was not the key determinant factor for cell yields of the outer thigh region, whereas high negative pressure had a positive influence on the cell yields of the abdomen region. However, for the subsequent culturing, no significant relationship was identified between the characteristics of isolated ADSCs and the level of negative pressure used during liposuction. In addition, the harvesting site influenced the ADSCs only mildly in some parameters on specific days of the culture (i.e., diameter on day 1). In general, no significant influence of the harvesting site was observed on the cell number, mitochondrial activity, viability, diameter, or on the presence of CD markers. These thigh ADSCs reached a higher cell number than for abdomen ADSCs on day 7 only in cases where cells from the inner thigh and outer thigh areas were evaluated together. However, we observed donor-to-donor variability in initial adhesion, in absolute cell numbers, and in the expression of some CD markers. Thus, our results could suggest that donor-to-donor differences may be affected not only by the harvesting site and by negative pressure but also by other factors. For subsequent *in vitro* culturing and use in tissue engineering, it seems that the harvesting site and the level of negative pressure do not have a crucial or limiting effect on basic ADSC characteristics. Nevertheless, it is necessary to make a thorough investigation of the area from which ADSCs are to be harvested and the specific liposuction procedure that is to be used, with reference to the purpose for which the adipose tissue is being harvested.

## Figures and Tables

**Table 1:** Donors included in our study. The group of females (*n* = 14) and one male (*n* = 1; abdomen site) underwent tumescent liposuction, in which adipose tissue was harvested from the inner thigh (*n* = 3), from the outer thigh (*n* = 7), and from the abdomen (*n* = 9). In each harvesting site, the lipoaspirate was obtained both under low and under high negative pressure.

Donor site	Age (years)	BMI (kg/m^2^)	No. of samples
Inner thigh	42.0 ± 4.6	27.70 ± 7.40	3
Outer thigh	35.4 ± 7.8	23.56 ± 2.45	7
Abdomen	38.3 ± 8.6	25.06 ± 4.08	9
Together	37.8 ± 7.8	25.44 ± 4.37	19 samples from 15 donors

**Table 2:** The viability of ADSCs. The viability of ADSCs harvested under low pressure and under high pressure from the inner thigh area, from the outer thigh area, and from the abdomen area on days 1, 3, and 7 in passage 2. No significant difference was observed between the outer thigh and the abdomen harvesting sites. The inner thigh region was not statistically compared to the outer thigh and abdomen regions due to the relatively small sample size.

Group of cells	Viability of ADSCs (%)					
	Day 1		Day 3		Day 7	
	Median	IQ range	Median	IQ range	Median	IQ range
Low I thigh	91.7	90.5-94.6	96.2	93.1-96.5	93.2	92.7-95.8
High I thigh	91.9	89.9-93.2	94.8	92.7-95.3	90.3	88.6-94.6
Low O thigh	93.6	84.8-95.8	93.8	91.2-96.5	95.2	89.4-96.9
High O thigh	93.5	80.7-94.5	96.6	94.3-97.3	95.9	95.6-97.0
Low abdomen	88.0	87.5-90.0	94.6	90.8-95.3	94.8	91.7-97.1
High abdomen	92.4	90.3-93.7	93.5	92.4-95.0	95.2	93.2-97.0

**Table 3:** The diameter of ADSCs. The diameter of ADSCs was measured using the Vi-CELL XR Cell Counter on days 1, 3, and 7; *p* < 0.05 (^∗^) is for harvesting area significance testing (i.e., outer thigh and abdomen). The inner thigh region was not statistically compared to the outer thigh and abdomen regions due to the relatively small sample size.

Group of cells		Diameter of ADSCs (microns)		
		Day 1	Day 3	Day 7
		Mean ± SD	Mean ± SD	Mean ± SD
Low I thigh		16.76 ± 0.74	14.67 ± 0.13	12.55 ± 0.38
High I thigh		15.73 ± 0.39	14.92 ± 0.26	12.91 ± 1.18
Low O thigh	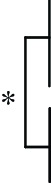	16.32 ± 0.82	14.71 ± 1.46	12.97 ± 0.49
High O thigh		17.04 ± 0.80	14.52 ± 1.10	13.77 ± 0.68
Low abdomen		15.67 ± 1.33	14.52 ± 1.48	13.39 ± 0.87
High abdomen		15.70 ± 1.27	14.93 ± 1.43	13.86 ± 1.10

**Table 4:** The cell mitochondrial activity of ADSCs. The cell mitochondrial activity of ADSCs measured on days 3 and 7. No significant difference was observed between the outer thigh and the abdomen harvesting sites. The inner thigh region was not statistically compared to the outer thigh and abdomen regions due to the relatively small sample size.

Group of cells	Cell mitochondrial activity (absorbance)	
	Day 3	Day 7
	Mean ± SD	Mean ± SD
Low I thigh	0.38 ± 0.25	0.41 ± 0.22
High I thigh	0.34 ± 0.29	0.41 ± 0.31
Low O thigh	0.53 ± 0.17	0.69 ± 0.14
High O thigh	0.52 ± 0.24	0.62 ± 0.10
Low abdomen	0.51 ± 0.25	0.71 ± 0.29
High abdomen	0.47 ± 0.24	0.69 ± 0.29

**Table 5:** The percentage of CD surface markers in ADSCs. The percentage of CD105-, CD90-, CD73-, CD29-, CD146-, CD45-, CD31-, and CD34-positive ADSCs. No significant difference was observed between the outer thigh and the abdomen harvesting sites. The inner thigh region was not statistically compared to the outer thigh and abdomen regions due to the relatively small sample size.

Group of cells	CD markers (% positive cells)							
	CD105		CD90		CD73		CD29	
	Median	IQ range	Median	IQ range	Median	IQ range	Median	IQ range
Low I thigh	99.9	99.2-99.9	99.5	99.5-99.7	99.9	99.8-100	99.8	99.2-100
High I thigh	99.9	94.1-99.9	99.6	99.3-99.8	99.9	99.9-100	99.8	99.8-100
Low O thigh	99.9	98.3-100	99.6	99.2-99.9	100	99.9-100	99.8	99.8-100
High O thigh	99.9	96.2-99.9	99.6	99.2-99.9	100	99.9-100	99.9	99.8-100
Low abdomen	99.5	82.3-99.9	99.4	97.5-99.8	99.8	99.6-99.9	99.6	90.5-99.8
High abdomen	98.9	89.1-99.8	99.5	97.3-99.8	99.8	99.6-99.9	99.6	95.1-99.8

Group of cells	CD markers (% positive cells)							
	CD146		CD45		CD31		CD34	
	Median	IQ range	Median	IQ range	Median	IQ range	Median	IQ range
Low I thigh	3.9	2.9-4.5	4.3	3.9-4.7	0.5	0.3-1.0	0.4	0.3-0.7
High I thigh	6.0	2.5-7.4	3.3	2.9-4.0	0.8	0.4-1.0	0.8	0.4-1.7
Low O thigh	3.9	1.3-5.2	1.8	1.5-6.9	0.5	0.2-0.7	1.1	0.4-6.3
High O thigh	5.4	2.7-24.6	1.8	1.1-12.6	0.6	0.1-2.4	0.9	0.3-6.1
Low abdomen	10.9	3.4-35.4	5.2	4.5-7.5	0.3	0.2-0.5	1.0	0.5-1.6
High abdomen	4.7	2.6-28.5	4.1	3.1-5.4	0.4	0.2-1.0	0.9	0.5-1.7

## Data Availability

Data can be requested from the corresponding author on reasonable request.

## Abbreviations

ADSC: Adipose tissue-derived stromal cell
BMSC: Bone marrow mesenchymal stromal cell
BMI: Body mass index
BSA: Bovine serum albumin
DMEM: Dulbecco's modified Eagle medium
DT: Doubling time
FBS: Foetal bovine serum
FGF2: Fibroblast growth factor basic
IQ range: Interquartile range
MSC: Mesenchymal stromal cell
SVF: Stromal vascular fraction.
